# Conductive-bridging random access memory: challenges and opportunity for 3D architecture

**DOI:** 10.1186/s11671-015-0880-9

**Published:** 2015-04-18

**Authors:** Debanjan Jana, Sourav Roy, Rajeswar Panja, Mrinmoy Dutta, Sheikh Ziaur Rahaman, Rajat Mahapatra, Siddheswar Maikap

**Affiliations:** Thin Film Nano Tech. Lab., Department of Electronic Engineering, Chang Gung University, 259 Wen-Hwa 1st Rd., Kwei-Shan, Tao-Yuan 333 Taiwan; Department of Electronics and Communication Engineering, National Institute of Technology, Durgapur, 713 209 India

**Keywords:** CBRAM, Conductive bridge, Resistive switching, Chalcogenide, Solid electrolyte, Bilayer, Three-dimensional (3D), Memory

## Abstract

The performances of conductive-bridging random access memory (CBRAM) have been reviewed for different switching materials such as chalcogenides, oxides, and bilayers in different structures. The structure consists of an inert electrode and one oxidized electrode of copper (Cu) or silver (Ag). The switching mechanism is the formation/dissolution of a metallic filament in the switching materials under external bias. However, the growth dynamics of the metallic filament in different switching materials are still debated. All CBRAM devices are switching under an operation current of 0.1 μA to 1 mA, and an operation voltage of ±2 V is also needed. The device can reach a low current of 5 pA; however, current compliance-dependent reliability is a challenging issue. Although a chalcogenide-based material has opportunity to have better endurance as compared to an oxide-based material, data retention and integration with the complementary metal-oxide-semiconductor (CMOS) process are also issues. Devices with bilayer switching materials show better resistive switching characteristics as compared to those with a single switching layer, especially a program/erase endurance of >10^5^ cycles with a high speed of few nanoseconds. Multi-level cell operation is possible, but the stability of the high resistance state is also an important reliability concern. These devices show a good data retention of >10^5^ s at >85°C. However, more study is needed to achieve a 10-year guarantee of data retention for non-volatile memory application. The crossbar memory is benefited for high density with low power operation. Some CBRAM devices as a chip have been reported for proto-typical production. This review shows that operation current should be optimized for few microamperes with a maintaining speed of few nanoseconds, which will have challenges and also opportunities for three-dimensional (3D) architecture.

## Review

### Background

In recent days, resistive random access memory (RRAM) technology is one of the most promising and reliable alternative solutions to overcome the scaling bottleneck of FLASH [1]. The advantages of RRAM devices are their simple metal-insulator-metal structure, low fabrication cost, long endurance, and non-volatile properties with low power consumption, multi-level cell (MLC) operation, and especially excellent scaling below the <11 nm technology node [2-5]. One of the RRAM devices is conductive-bridging random access memory (CBRAM), which has high possibility to fulfill the requirements for next-generation non-volatile memory (NVM) technology as mentioned in the International Technology Roadmap for Semiconductors (ITRS) [1]. In addition, major benefits are high speed (few nanoseconds) and low voltage operation (±3 V). Basically, the structure of CBRAM devices consists of one metal electrode which is electrochemically active (i.e., anode) or oxidized under external positive bias, such as Ag or Cu, and another one which is electrochemically inert (i.e., cathode), such as platinum (Pt), iridium (Ir), gold (Au), tungsten (W), or titanium-nitride (TiN). These two electrodes are separated by a solid electrolyte or oxide materials. In 1976, Hirose et al. [6] reported memory switching using a Ag dendrite in a Ag-doped As_2_S_3_ film in a Ag/As_2_S_3_/Mo structure. In 1999, Kozicki et al. [7] reported a programmable metallization cell (PMC) device using metal-doped chalcogenide films. PMC devices have different names like electrochemical metallization cell (ECM), ‘atom switch’ [8], or CBRAM [9]. Generally, they are called ‘CBRAM’. According to their concepts, when a positive voltage is applied on the anode, an electrochemical reaction occurs, which oxidizes the metal to form ions (Cu^*z*+^, *z* = 1, 2, or Ag^+^). The cations drift through the solid electrolyte switching layer under the electric field, and the metal ions are reduced on the inert electrodes. As this process continues, a metallic filament is established in between the two electrodes and the device switches from the high resistance state (HRS) to the low resistance state (LRS). By changing the polarity of the voltage, an electrochemical dissolution of the conductive bridges takes place, resetting the device from the LRS to the HRS. The resistance state changes by applying external bias, and the switching is reversible. It is true that device reliability including stable switching characteristics, program/erase endurance, and data retention at 85°C for 10 years at a low current of few microamperes is a very challenging issue for production. In addition, the switching mechanism in different structures including materials and electrodes is not understood clearly. Especially, the growth kinetics of the metallic filament in oxide- and non-oxide-based materials are still debated. Although the memory performances of a designed structure with low operation current have huge opportunities to fulfill the requirements of ITRS, they are not achieved yet. Even though many papers have been reported by several groups, a complete review on CBRAM performances including switching characteristics, reliability, mechanism, and so on for real production is needed, which is not reported yet.

Here, the challenges and opportunities of CBRAM devices using different switching materials such as chalcogenides, oxides, and bilayers in different structures have been reviewed. Memory performances such as switching characteristics, endurance, multi-level cell operation, and data retention have been discussed. Memory devices can be operated with an operation current of 0.1 μA to 1 mA under an operation voltage of ±2 V. Filament growth dynamics in different switching materials have been discussed. Device reliabilities such as switching uniformity, endurance, and data retention under a few microamperes are very challenging issues. The device can be scaled down below the 11 nm technology node and has a very good opportunity for non-volatile crossbar memory for 3D architecture in the near future. Finally, CBRAM as a chip has been discussed for future production.

### Materials and deposition methods

CBRAM performance depends on switching materials including different non-oxides and oxides. The non-oxides, i.e., chalcogenides, are GeSe_*x*_ [10-12], GeS_2_ [13,14], GeTe [15], Cu_2_S [16], Ag_2_S [8], and so on. The oxide-based materials are Ta_2_O_5_ [16,17], SiO_2_ [18], ZrO_2_ [19], GeO_*x*_ [20,21], and so on. Other materials such as amorphous Si [22] and Si_3_N_4_ [23] have been reported also. Some reported results show CBRAM characteristics using bilayer switching materials: one layer is used as a buffer or interfacial layer and the other one is used as a switching layer. Bilayers such as Cu-Te/GdO_*x*_ [24], Cu-Te/SiO_*x*_ [25], MoO_*x*_/GdO_*x*_ [26], TiO_*x*_/TaSiO_*y*_ [27], GeSe_*x*_/TaO_*x*_ [28], Ti/TaO_*x*_ [29], Cu-Te/Al_2_O_3_ [30], TiW/Al_2_O_3_ [31], and CuTe-C/Al_2_O_3_ [32] have been reported by many groups. Deposition process parameters of different switching materials are discussed below.

**Figure 1:**
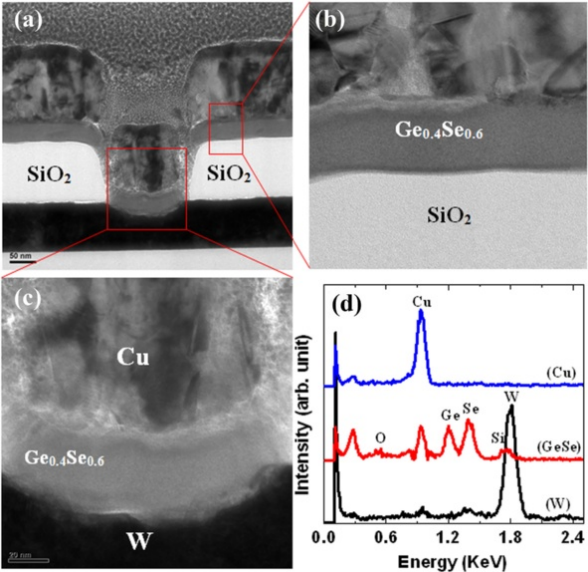
TEM images and EDX analysis of a Cu/Ge_0.4_Se_0.6_/W pristine memory device. **(a)** TEM image of a Cu/Ge_0.4_Se_0.6_/W pristine memory device with a scale bar of 50 nm. The Ge_0.4_Se_0.6_ film shows to be amorphous, as shown in the **(b)** outside and **(c)** inside of the via-hole region. **(c)** Different layers are clearly shown by the dark-field TEM image and **(d)** corresponding EDX analysis of the W, Ge_0.4_Se_0.6_, and Cu films from **(a)** [33].

**Figure 2:**
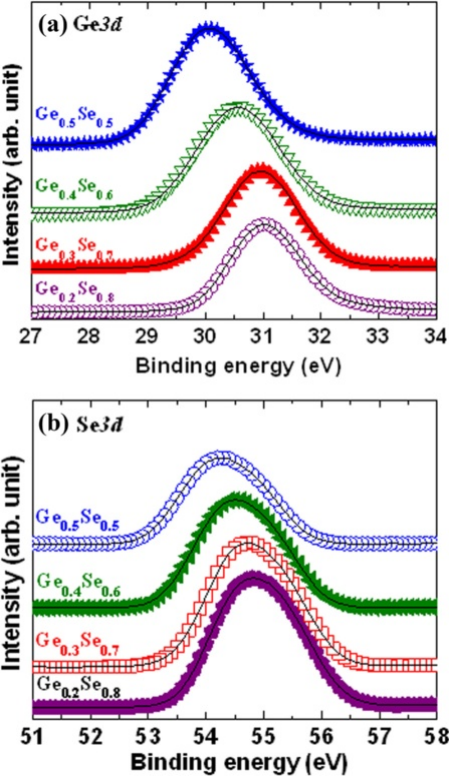
XPS spectra of Ge_*x*_Se_1 − *x*_ solid electrolytes for **(a)** Ge *3d* and **(b)** Se *3d* electrons. The binding energy increased with increasing Se contents.

Memory devices using chalcogenide-based switching materials are reported below. Chalcogenide glasses are based on the chalcogen elements, and they have a wide range of properties such as optical and electrical ones, as reported in the literature. Several types of chalcogenide-based solid electrolytes have been reported for future resistive switching memory applications. Kozicki et al. [10] reported a Ag/Ag_33_Ge_20_Se_47_/Ni PMC device. A 100-nm-thick Ni as a bottom electrode (BE) was fabricated on SiO_2_/Si substrates. Then, 50-nm-thick Ge_70_Se_30_ was evaporated with the help of a Knudsen-type cell under 10^−6^ Torr vacuum. This approach will help in maintaining almost the same level of elementary content of the switching material. A very low deposition rate of 0.03 nm/s was maintained to enhance the step coverage and filled into the narrow via-holes. Immediately after that, a 30-nm-thick Ag film was deposited to form a Ag:GeSe solid electrolyte. In this case, Ag diffusion by exposure to 405-nm ultraviolet (UV) radiation was time consuming and cost effective. To avoid this issue, Schindler et al. [11] reported a Ag/Ag-GeSe/Pt structure. Their memory cells consisted of a 100-nm-thick continuous Pt BE on SiO_2_/Si substrates. A Si_3_N_4_ layer was deposited on Pt by plasma-enhanced chemical vapor deposition (PECVD) method with a deposition rate of 0.2 nm/s. Via-holes with varying diameters from 2.5 to 50 μm were patterned by optical lithography. The solid electrolyte layer was 50-nm-thick Ge_0.3_Se_0.7_ and was deposited by radio-frequency sputtering or physical vapor deposition (PVD) at a rate of 0.2 nm/s. The applied power ranged from 5 to 25 W. The top electrodes were patterned by optical lithography. Then, the Ag layer was deposited by thermal evaporation at a deposition rate of 0.5 nm/s. After that, a lift-off process was carried out to obtain the final device. Due to the low melting point of Se (221°C), it is difficult to control the composition of GeSe after deposition by sputtering. To keep the same composition, the GeSe material was deposited by using an electron beam evaporator, which we have also reported previously [12]. The resistive switching material and electrodes were deposited by PVD processes such as sputtering, electron beam evaporation, and thermal evaporation. Initially, 8-in. p-type Si (100) wafers were used to deposit a SiO_2_ layer with a thickness of approximately 200 nm. The SiO_2_ layer was grown by thermal oxidation. Then, W or TiN metal was used as a BE on the SiO_2_/Si substrates. The thickness of the BE was approximately 200 nm. To design the memory devices, a SiO_2_ layer with a thickness of approximately 150 nm was deposited. Lithography was used to pattern via-holes with different nominal areas from 200 × 200 nm^2^ to 8 × 8 μm^2^ of the active switching region. After defining the via-hole regions, reactive-ion etching (RIE) process was used to etch the uncovered SiO_2_ films and to open the W BE contact. Following this, a Ge_*x*_Se_1 − *x*_ solid electrolyte with a thickness of approximately 40 nm was deposited using an electron beam evaporator. Pure Ge_*x*_Se_1 − *x*_ (*x* = 0.2 to 0.5) granules (commercially available) with different compositions such as Ge_0.2_Se_0.8_, Ge_0.3_Se_0.7_, Ge_0.4_Se_0.6_, and Ge_0.5_Se_0.5_ were used for deposition. The chamber pressure was 5 × 10^−6^ Torr prior to deposition. The deposition rate was 1 to 2 Å/s. Then, a Cu top electrode (TE) with a thickness of approximately 40 nm was deposited using a thermal evaporator. A 160-nm-thick layer of Al was then deposited *in situ* using the same thermal evaporator. Otherwise, a 100- to 200-nm-thick Cu electrode was used. Finally, a lift-off process was performed to obtain the resistive switching memory device. To investigate the memory device and microstructure of the solid electrolyte films, transmission electron microscopy (TEM) was carried out using a FEI Tecnai G2F-20 field emission system with an energy of 200 kV. The memory devices for TEM observation were prepared using an FEI Helios-400s system with an operating voltage of 5 kV and using a Ga^+^ ion source. Figure 1a shows a TEM image and energy-dispersive X-ray spectroscopy (EDX) analysis of a Cu/Ge_0.4_Se_0.6_/W structure [33]. The thicknesses of the Cu and W electrodes are found to be 136 and 85 nm, respectively. The thickness of the Ge_0.2_Se_0.8_ solid electrolyte films is approximately 38 nm. A small via-hole size is about 140 nm in width, which is lower (slightly) than our design size (200 nm). The Ge_0.4_Se_0.6_ film is amorphous, as shown in the outside (Figure 1b) and inside (Figure 1c) of the via-hole regions. A layer-by-layer structure is observed from those images. All materials such as Cu, Ge, Se, and W in the Cu TE, GeSe switching, and W BE layers are confirmed by EDX analysis (Figure 1d). Figure 2a shows the X-ray photo-electron spectroscopy (XPS) spectra of the Ge_*x*_Se_1 − *x*_ solid electrolyte films. The peak binding energies of Ge_0.5_Se_0.5_, Ge_0.4_Se_0.6_, Ge_0.3_Se_0.7_, and Ge_0.2_Se_0.8_ films are found to be 30.1, 30.6, 31, and 31.1 eV, respectively. The peak binding energy of Ge *3d* electrons increased (30.1 to 31.1 = 1 eV) owing to increased Se content [34,35]. The chemical shifts of Ge *3d* (29 eV) electrons are 1.1, 1.6, 2.0, and 2.1 eV for Ge_0.5_Se_0.5_, Ge_0.4_Se_0.6_, Ge_0.3_Se_0.7_, and Ge_0.2_Se_0.8_ films, respectively. A similar chemical shift of Ge *3d* electrons was reported by Ueno et al. [35]. According to their reported results, the chemical shifts of Ge *3d* electrons are 2.2 ± 0.1 eV for GeSe_2_ and 1.7 ± 0.1 eV for GeSe, while those of Se *3d* electrons are negative, which is similar to our results. The corresponding binding energies of Se *3d* core-level electrons for Ge_0.5_Se_0.5_, Ge_0.4_Se_0.6_, Ge_0.3_Se_0.7_, and Ge_0.2_Se_0.8_ films are found to be 54.2, 54.5, 54.7, and 54.8 eV, respectively (Figure 2b). A positive shift of binding energies from 54.2 to 54.8 = 0.6 eV is owing to the higher Se contents. The chemical shifts of Se *3d* (55.2 eV) electrons are found to be −1.0, −0.7, −0.5, and −0.4 eV for Ge_0.5_Se_0.5_, Ge_0.4_Se_0.6_, Ge_0.3_Se_0.7_, and Ge_0.2_Se_0.8_ films, respectively. However, the chemical shifts of Se *3d* (55.5 eV) electrons are −1 eV for GeSe_2_, as reported by Ueno et al. [35]. Slight variation of binding energies is related to the material deposition process and conditions [36]. On the other hand, the O *1s* core-level electron signal is not observed in the Ge_*x*_Se_1 − *x*_ film. To search better thermal stability, other switching materials are discussed below.

Kozicki et al. [13] also reported the fabrication of a Ag (or Cu)/GeS/W structure. They deposited a W BE on SiO_2_/Si substrates by chemical vapor deposition (CVD) method and then covered it with a 100-nm-thick SiO_2_ layer. Via-holes with different diameters ranging from 180 nm to 5 μm were opened on the SiO_2_ layer by optical projection lithography and RIE. Then, 40- and 50-nm-thick Ge_*x*_S_1 − *x*_ (0.30 < *x* < 0.35) films were deposited by PVD. Next, a 25-nm-thick Ag (or Cu) layer was deposited by PVD. The chamber pressure was maintained at 10^−6^ Torr. It is important to note that the deposition of the switching layer is a difficult process as the vapor pressure of the S component is very high. Vianello et al. [14] reported CBRAM devices using a Ag/Sb:GeS_2_/W-plug structure, and 1T1R configuration in 8 × 8 arrays integrated in the complementary metal-oxide-semiconductor (CMOS) process was done. The plug size was 0. 2 μm. The thickness of the Ag-doped GeS_2_ layer, which was deposited by co-sputtering, was 30 nm. The Sb material was doped 2% to 20% in GeS_2_. A thin Ag layer was dissolved into GeS_2_ using a photo-diffusion process [13]. Then, the Ag layer as a TE was deposited. Choi et al. [15] reported a Cu/GeTe/TaN structure. A SiO_2_ layer with a thickness of 100 nm was grown on a Si substrate. Corresponding materials such as TaN, GeTe, and SiO_2_ were deposited (in the given order) onto the prepared substrate using a radio-frequency (RF) sputtering system. The thicknesses of TaN, GeTe, and SiO_2_ layers were 30, 50, and 100 nm, respectively. Via-hole devices with different sizes of 200 nm, 400 nm, 10 μm, and 50 μm were patterned by e-beam lithography. Banno et al. [16] reported a Cu/Cu_2 *− α*_S/Pt structure. Terabe et al. [8] reported a gap-type resistive switching memory device. At first, an Ag_2_S-coated Ag wire was crossed by a Pt wire. They mentioned that the ‘crossbar’ structure was convenient for integrating switches to be used in actual devices. Some oxide-based materials for CBRAM devices are explained below.

**Figure 3:**
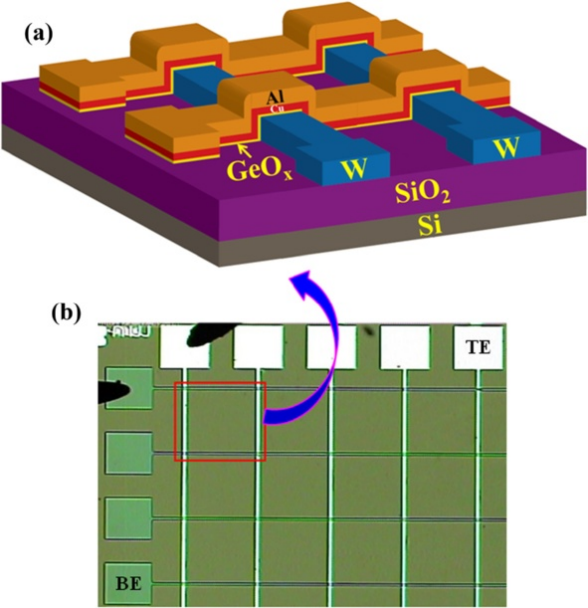
Schematic illustration **(a)** and optical image **(b)** of the cross-point memory devices for 3D architecture. The area of the active cross-point is approximately 1.2 × 1.2 μm^2^. The thickness of the GeO_*x*_ solid electrolyte film is approximately 10 nm [21].

Banno et al. [16] reported a Cu/Ta_2_O_5_/Pt structure for their CBRAM devices. A 15-nm-thick Ta_2_O_5_ film with an O/Ta ratio of 2.5 was deposited on a Pt electrode by RF sputtering. Next, a 50-nm-thick Cu electrode was deposited and covered by a 50-nm-thick Pt film to avoid oxidization of Cu. In our previous work [17], we have fabricated a Cu/Ta_2_O_5_/W structure. The via-holes were patterned as mentioned above in our work. Then, 15- to 20-nm-thick Ta_2_O_5_ films were deposited using an e-beam evaporator. The Ta_2_O_5_ granules were used. Next, a Cu TE with a thickness of 100 nm was deposited using a thermal evaporator. Finally, a lift-off process was done to obtain the memory devices. A post metal annealing treatment was performed at a temperature of 350°C for 1 min in N_2_ ambient. Schindler et al. [18] reported a Cu/SiO_2_/W structure. A 100-nm-thick W BE was deposited by CVD on SiO_2_/Si substrates. Then, a SiO_2_ switching material with thicknesses varying from 12 to 50 nm was grown by electron beam evaporation. A Cu TE with a thickness of 35 to 45 nm was deposited. Then, Cu was diffused into SiO_2_ by thermal annealing at 610°C to have a Cu/Cu:SiO_2_/W structure. Li et al. [19] fabricated a Ag/ZrO_2_/Au structure. First, a 50-nm-thick Ag layer was deposited using an electron beam evaporator. A ZrO_2_ film was also deposited. The vacuum pressure and the deposition rate were 2 × 10^−6^ Torr and 1 Å/s, respectively. After that, a 50-nm-thick Au TE with different areas such as 100 × 100 μm^2^ to 800 × 800 μm^2^ was deposited. A GeO_*x*_ switching material as CMOS compatible was reported by us [20]. Via-holes with size ranging from 0.2 × 0.2 μm^2^ to 8 × 8 μm^2^ were reported. A GeO_*x*_ film with a thickness of approximately 10 nm was then deposited by RF sputtering. The GeO_*x*_ film showed to be polycrystalline. The chamber pressure was 2 × 10^−5^ Torr. The deposition power and pressure were 50 W and 2 × 10^−2^ Torr, respectively. Argon (Ar) gas with a flow rate of 25 sccm was used. A Ge target was used to deposit the GeO_*x*_ solid electrolyte. Then, a Cu TE with a thickness of approximately 200 nm was deposited using a thermal evaporator. A lift-off process was next performed to obtain the final via-hole devices in a Cu/GeO_*x*_/W structure. Cross-point memory devices using an Al/Cu/GeO_*x*_/W structure were also reported [21]. First, a SiO_2_ layer with a thickness of approximately 200 nm was grown by a wet oxidation process. After the masking process, a GeO_*x*_ film with a thickness of 10 nm was deposited by the same RF sputtering. A Cu layer with a thickness of 40 nm was deposited using a thermal evaporator, and then, an Al layer with a thickness of approximately 160 nm was deposited *in situ* using a thermal evaporator. Finally, a lift-off process was performed to get the cross-point devices. A schematic illustration of the fabricated GeO_*x*_-based cross-point memory device is shown in Figure 3a [21]. The GeO_*x*_ solid electrolyte was sandwiched in between Cu and W electrodes. An optical micrograph (OM) image of the 5 × 5 cross-points is shown in Figure 3b. The memory characteristics of this cross-point memory device are discussed later. Kim et al. [22] reported CBRAM characteristics using a Ag/a-Si/SiGe/W crossbar structure. Initially, a W BE with a thickness of approximately 20 nm was deposited by sputtering. In the next step, a boron-doped Si_*x*_Ge_1 − *x*_ film of 20 nm was deposited by thermal CVD at 425°C. An amorphous Si (a-Si) as a resistive switching material was deposited by PECVD method. The thickness of the a-Si film was approximately 20 nm. Finally, a Ag TE was deposited. This complete stack was patterned by electron beam lithography and plasma etching process. The thickness of all the layers was 50 nm half-pitch arrays. Sun et al. [23] also reported bipolar CBRAM characteristics using a Ag/Si_3_N_4_/Pt memory device. The device size was 100 × 100 μm^2^. Even though many groups reported a single-layer switching material, bilayer switching materials showed to be promising.

**Figure 4:**
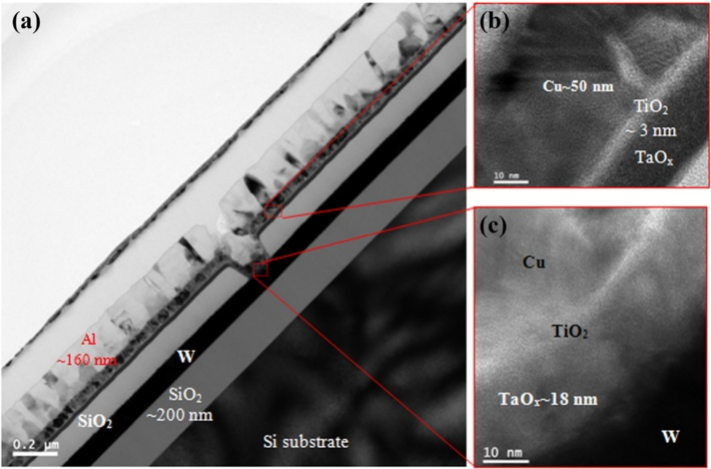
TEM image of an Al/Cu/Ti/TaO_*x*_/W structure. **(a)** HRTEM images of the **(b)** outside and **(c)** inside of the via-hole device, as shown in **(a)**. After deposition of a Ti nanolayer, it became TiO_2_ [29].

Aratani et al. [24] investigated CBRAM characteristics using a TE/Cu-Te/GdO_*x*_/W structure. In this case, both TE and switching materials were deposited on the BE by a sputtering process. Device sizes of 20 and 50 nm were designed. The Cu-Te layer behaved as a buffer layer, and the GdO_*x*_ layer was used as a switching layer. The Cu filament formation/dissolution in the GdO_*x*_ layer was controlled by a buffer layer through external bias. Similarly, Cu-Te/SiO_*x*_ bilayers were also reported [25]. Yoon et al. [26] reported MoO_*x*_/GdO_*x*_ bilayers in a Cu/MoO_*x*_/GdO_*x*_/Pt structure. A 10-nm-thick GdO_*x*_ layer was deposited on a Pt/Ti/SiO_2_/Si substrate using an electron beam evaporator. A 100-nm-thick Pt was also deposited. After a lift-off process, a device size of 50 × 50 μm^2^ was fabricated. A NanoBridge CBRAM device was reported by using a Cu/Ta/TaSiO_*y*_/Ru structure [27]. The memory cell was integrated in the CMOS process. Via-holes with cell sizes ranging from 1 μm to 50 nm were completed. After deposition of a Ti or Ta barrier layer on Cu, a TaSiO_*y*_ solid electrolyte was deposited by RF sputtering in Ar*/*O_2_ gas. The CBRAM devices were connected through contact holes and controlled the switching through a CMOS transistor. Therefore, the 1T1R configuration was developed. In our previous work [28], we reported GeSe_*x*_/TaO_*x*_ bilayer materials in an Al/Cu/GeSe_*x*_/TaO_*x*_/W structure. Although this CBRAM device using GeSe_*x*_/TaO_*x*_ bilayers shows promising resistive switching characteristics, the thermal stability of the Ge_20_Se_80_ material is an issue. To have a CMOS-compatible structure, we reported a Al/Cu/TiO_2_/TaO_*x*_/W CBRAM device [29]. The via-hole devices were fabricated as follows [17,28]. A Ta_2_O_5_ film with a thickness of 18 nm was deposited from pure Ta_2_O_5_ granules using an electron beam evaporator. The resulting Ta_2_O_5_ film was mixed with Ta metal (i.e., TaO_*x*_, where *x* < 2.5), as characterized by XPS. The Cu as mobile ions plays a major role in the Al/Cu/TaO_*x*_/W structure resistive switching memory device. The Cu can be oxidized at the Cu/TaO_*x*_ interface during deposition and hinders the resistive switching memory performance. Therefore, Cu oxidation is expected to be avoided by inserting a Ti nanolayer at the Cu/TaO_*x*_ interface. A thin Ti layer with a thickness of approximately 3 nm was deposited *in situ* using an electron beam evaporator with Ti granules. Finally, a device with an Al/Cu/TiO_2_/TaO_*x*_/W structure was obtained. These memory devices were annealed by rapid thermal annealing (RTA) at 350°C in N_2_ ambient for 1 min. Oxygen accumulated in the Ti nanolayer and formed TiO_2_. Figure 4a shows a TEM image of the device with a Ti nanolayer at the Cu/TaO_*x*_ interface [29]. The device size was approximately 150 × 150 nm^2^. All layers were covered well at the active and outer regions (Figure 4b,c). Oxygen accumulated in the Ti layer as expected, resulting in a thin TiO_2_ layer of approximately 3 nm. This accumulated oxygen originated from the TaO_*x*_ layer and formed a more defective TaO_*x*_ film, which is in agreement with the Gibbs free energy and helps to have repeatable resistive switching characteristics. On the other hand, Al_2_O_3_-based bilayer materials were also reported. Goux et al. [30] reported the compositional effect of a Cu-Te buffer layer on the switching characteristics of a Pt/Cu_*x*_Te_1 − *x*_/Al_2_O_3_/Si structure. The Al_2_O_3_ layer with a thickness of 3 nm was deposited by atomic layer deposition (ALD). Using a shadow mask, Cu_*x*_Te_1 − *x*_ dots were deposited by using a co-sputtering system. Cu and Cu_0.1_Te_0.9_ targets were used. The size of the dots was 3 mm wide and 50 nm thick. Belmonte et al. [31] fabricated a Cu/TiW/Al_2_O_3_/W structure in a 90 nm technology node. Firstly, a 3-nm-thick Al_2_O_3_ layer was deposited on a W BE by ALD. After that, a 10-nm-thick TiW alloy with 25 at % of Ti was deposited by using a sputtering system. Then, a Cu seed layer was deposited by a sputtering process. At last, a TiN TE was deposited to get the final device. Devulder et al. [32] reported a TiN/Cu_0.6_Te_0.4_-C/Al_2_O_3_/Si structure to enhance the thermal stability and switching behavior of memory devices. Table 1 presents a brief description about the material, structure, and fabrication procedure of CBRAM devices. Using chalcogenides, oxides, and bilayer materials in the above structures, the resistive switching memory characteristics are explained below.

**Table 1:** **CBRAM material and deposition methods**

**Device structure (TE/switching material/BE)**	**Deposition methods**			**Device size (μm)**
	**TE**	**Switching material**	**BE**	
Ag/Ag_33_Ge_20_Se_47_/Ni [10]	-	Evaporation	-	40, 75
Ag/Ag-GeSe/Pt [11]	-	RF sputtering	Thermal evaporation	2 to 50
Al/Cu/Ge_0.5_Se_0.5_/W [12]	Thermal	Electron beam	Sputtering	0.2 to 8.0
Ag or Cu/GeS/W [13]	PVD	PVD	CVD	0.18 to 5
Ag/Sb:GeS_2_/W [14]	-	RF PVD	-	0.2
Cu/Ta_2_O_5_/Pt [16]	-	RF sputtering	-	-
Cu/Ta_2_O_5_/W [17]	Thermal evaporator	E-beam evaporation	Sputtering	0.2 to 8.0
Cu/Cu:SiO_2_/W [18]	-	E-beam evaporation	CVD	0.35 to 5
Ag/a-Si/SiGe/W [22]	-	CVD	Sputtering	0.05
Ag/Si_3_N_4_/Pt [23]	RF sputtering	PECVD	RF sputtering	10
Cu/Cu-Te/GdO_*x*_/W [24]	Sputtering	Sputtering	-	0.02, 0.05
Cu/Ta/TaSiO_*y*_/Ru [27]	-	PECVD	-	0.05 to 1
Al/Cu/Ti/TaO_*x*_/W [29]	Thermal	Electron beam	Sputtering	0.15
Cu-Te/Al_2_O_3_/Si [30]	Co-sputtering	ALD	-	-
Al/TiN/Cu/TiW/Al_2_O_3_/W [31]	-	ALD	Sputtering	0.09

### Memory characteristics

#### Chalcogenide-based materials

**Figure 5:**
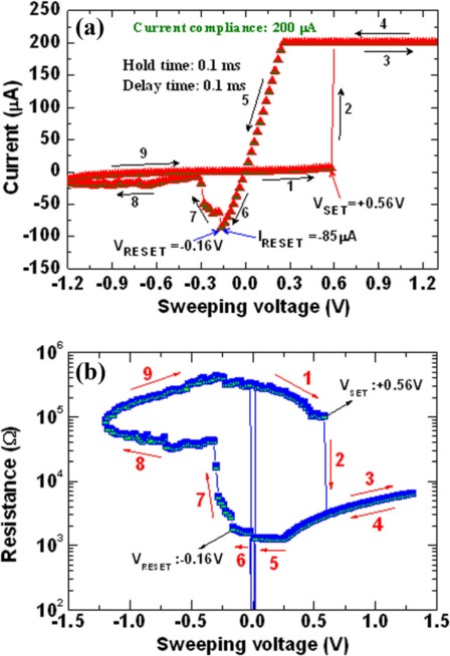
*I*-*V* and corresponding *R*-*V* hysteresis characteristics of an Al/Cu/Ge_0.4_Se_0.6_/W resistive memory device. **(a)** *I*-*V* hysteresis characteristics of an Al/Cu/Ge_0.4_Se_0.6_/W resistive memory device. The device size was 0.2 μm. **(b)** Corresponding *R*-*V* hysteresis characteristics of the device [33].

**Figure 6:**
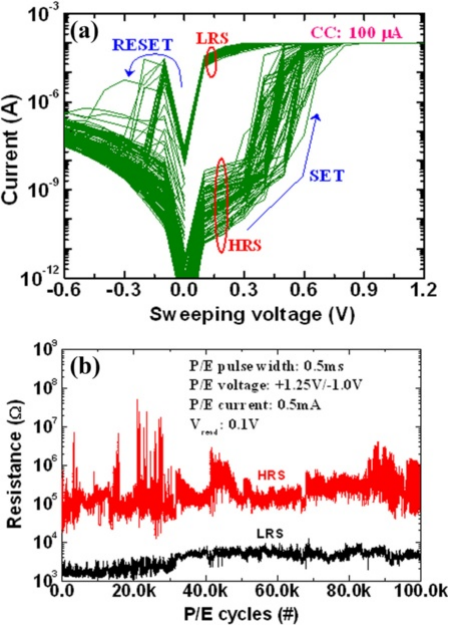
*I*-*V* hysteresis for a Cu/Ge_0.3_Se_0.7_/W memory device and typical P/E cycles of a Cu/Ge_0.2_Se_0.8_/W memory device. **(a)** *I*-*V* hysteresis of more than 100 consecutive DC cycles for a Cu/Ge_0.3_Se_0.7_/W memory device. The size of the memory device is 4 × 4 μm^2^. **(b)** Typical P/E cycles of a Cu/Ge_0.2_Se_0.8_/W memory device. Every P/E cycle was recorded. This suggests that the P/E cycles are more than 10^5^.

**Figure 7:**
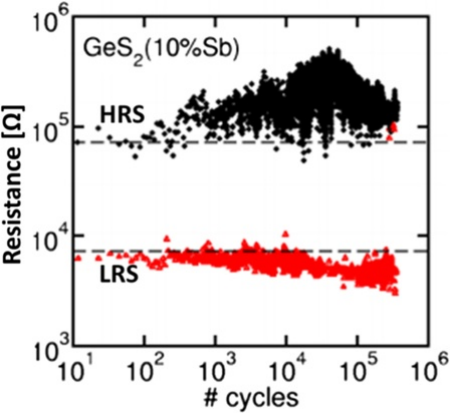
P/E endurance of >10^5^ cycles of Ag/Sb-doped GeS_2_/W-based 1S1R (one selector-one resistor configuration) CBRAM cell [14].

**Figure 8:**
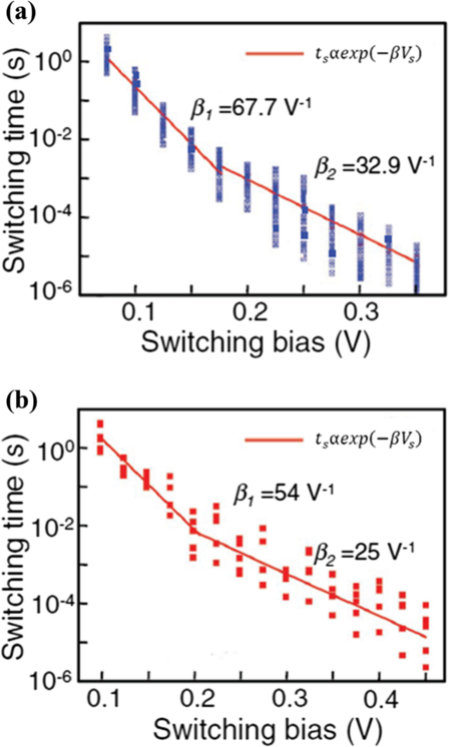
Switching time vs switching bias characteristics. For **(a)** Ag_2_S-based gap-type atomic switch at room temperature and **(b)** Cu_2_S-based gap-type atomic switch [38].

Germanium (Ge)-based chalcogenide glasses have been widely studied because of their well-known mechanism and also they offer several advantages, whereas Cu^*z*+^ or Ag^+^ ions exhibit high mobility. In our previous study [33], we have reported the bipolar current-voltage (*I*-*V*) characteristics of a Cu/Ge_0.4_Se_0.6_/W structure, as shown in Figure 5a. The step voltage was 20 mV during voltage sweep. The current compliance (CC) was 200 μA. The size of the memory device was 200 × 200 nm^2^. It is noted that the formation voltage is not required for Ge_*x*_Se_1 − *x*_ solid electrolyte-based CBRAM devices. The *I*-*V* hysteresis loop can be explained as follows by using arrows 1 to 9. Initially, a voltage applied to the TE is swept from 0 to +0.56 V (arrow 1); this is the HRS. Beyond the voltage of +0.56 V, there is an instantaneous switching from the HRS to the LRS (arrow 2). The resistive switching is observed beyond a voltage of 0.56 V, which is called SET voltage (*V*_SET_). To form a Cu metallic filament into the Ge_0.4_Se_0.6_ solid electrolyte, the applied bias should be larger than the SET voltage (arrow 3). The memory device keeps the LRS up to a negative voltage of −0.18 V (arrows 3 to 6). The resistive memory device reaches a HRS if the voltage applied is more than the negative voltage or RESET voltage (*V*_RESET_) < −0.16 V (arrow 7). A large RESET current (*I*_RESET_) of −85 μA confirms that the strong Cu metallic filament is formed into the Ge_0.4_Se_0.6_ solid electrolyte. To reach a HRS, a negative voltage of −1.2 V should be large enough (arrow 8). The metallic filament is gradually dissolved by applying a higher negative voltage. To get a second switching, the sweeping voltage can be followed using arrows 9 and 1. The corresponding resistance vs sweeping voltage (*R-V*) hysteresis characteristics are plotted in Figure 5b. A high resistance ratio (HRS/LRS) of approximately 3 × 10^6^ is observed, which is useful for MLC operation. Choi et al. [15] and Liaw et al. [37] also reported a high resistance ratio of >10^3^ to 10^5^. Kund et al. [9] have reported bipolar resistive switching phenomena using a Ag/GeSe/W structure at a CC of 2 μA. The *V*_SET_ was +0.2 V. Kozicki et al. [13] have reported resistive switching characteristics at a *V*_SET_ of +0.4 V and a CC of approximately 10 μA. One hundred consecutive *I-V* switching cycles at a CC of 100 μA are shown in Figure 6a. However, the step voltage was 0.1 V. The *V*_SET_ varied from 0.3 to 0.5 V. Variations of the resistance states and RESET current are also observed, due to the higher Se content (80%). Figure 6b shows the program/erase (P/E) cycles for every cycle. The P/E current and pulse width are 500 μA and 500 μs, respectively. The programming and erasing voltages are 1.25 and −1.0 V, respectively. The read voltage is 0.1 V. Even though large variations of resistance states are observed, the memory device will have 10^5^ P/E cycles. All memory devices using Ge_*x*_Se_1 − *x*_ solid electrolytes are performed more than >10^5^ P/E cycles, which is beneficial by using Ge_*x*_Se_1 − *x*_ solid electrolytes. Kozicki et al. [10] have reported >10^10^ read endurance using a Ag/Ag_33_Ge_20_Se_47_/Ag structure. Kund et al. [9] have investigated >10^6^ P/E endurance using a GeSe_*x*_-based CBRAM device. Vianello et al. [14] have also reported the P/E endurance characteristics of a Sb-doped GeS_2_-based chalcogenide-based CBRAM cell, as shown in Figure 7. The memory device performs >10^5^ P/E cycles at 30-ns pulse width with a resistance ratio of >10. However, there is voltage-time dilemma. For a high-speed operation, the program/erase voltage needs to be higher. Hasegawa et al. [38] have studied the switching time of a Ag_2_S- and Cu_2_S-based gap-type atomic switch as a function of bias voltage measured at room temperature, as shown in Figure 8. The switching time (*t*_s_) is expressed as function of switching bias (*V*_s_ > *V*_SET_), as shown in Equation 1:

1 $$ {t}_{\mathrm{s}}\infty \exp \left(-\beta {V}_{\mathrm{s}}\right) $$

**Figure 9:**
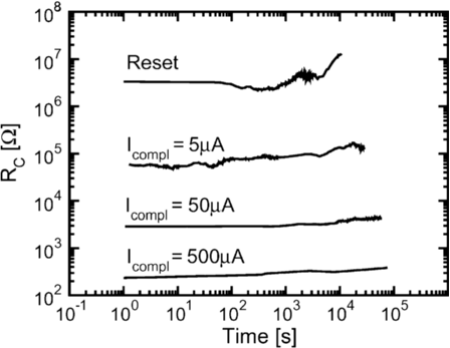
Excellent multi-level data retention of >10^5^ s. With varying *I*_compl_ (CC) from 5 to 500 μA of memory cell which is very promising for high-storage multi-bit operation [42].

**Figure 10:**
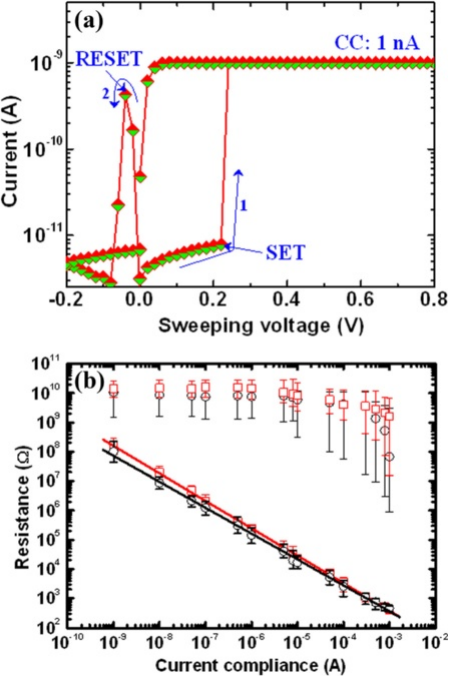
*I*-*V* hysteresis for Ge_0.2_Se_0.8_ and LRS/HRS vs CCs. **(a)** *I*-*V* hysteresis at a CC of 1 nA for a Ge_0.2_Se_0.8_ solid electrolyte. **(b)** LRS/HRS vs CCs for lower and higher Ge contents of the Ge_*x*_Se_1 − *x*_ solid electrolytes.

The exponential nature of the time vs voltage characteristics suggests that a higher SET voltage can drastically reduce the time to SET the memory cell. Even a 0.1-V increment can result in around one to two orders of reduction in SET time. The switching time can be distinguished into two decay components when the bias voltage is increased (Figure 8a). The values of decay factors *β*1 and *β*2 are 67.7 and 32.9 for low-voltage and high-voltage regions, respectively. Similar phenomena are also reported in a Cu_2_S switching material, as shown in Figure 8b. In this case, the values of decay factors *β*1 and *β*2 are 54 and 25 for low-voltage and high-voltage regions, respectively. These two exponential decay factors exhibit different rate limiting processes for the low-voltage and high-voltage regions. It is well known that there are only few mechanisms such as metallic filamentary switching that require few nanoseconds (<10 ns) to switch a device from SET to RESET and vice versa. Thus, for the 10-year data retention criterion, the device needs to maintain the ratio of the retention time to the SET time of around 10^16^ and *V*_SET_/*V*_read_ of around 10. Again, the read voltage needs to be high enough to maintain the ratio of *V*_program_/*V*_read_ at 10 for circuit constraint. It is also required that the cell withstand the read voltage for 10 years. Schindler et al. [39] have also reported this switching time-voltage dependence of SiO_2_-based CBRAM cells. Therefore, CBRAM devices can be operated at low current and stable data retention is also an important part. We have reported data retention characteristics of >11 h at a CC of 200 μA for a Cu/Ge_0.4_Se_0.6_/W CBRAM device [40]. The memory device shows data retention at higher temperatures (85°C and 150°C) and maintains a large resistance ratio of >100; however, long-time data retention does not show to be stable. Vianello et al. [14] have shown a data retention of >10^5^ s at 130°C using a Sb-doped GeS_2_ solid electrolyte. Jameson et al. [41] have reported an excellent data retention of >10^6^ s at 200°C in a Ag/GeS_2_/W memory cell. In addition, MLC capability is also important for high-density memory application. Russo et al. [42] have reported multi-level data retention characteristics of >10^5^ s with CC (or *I*_compl_) varying from 5 to 500 μA, as shown in Figure 9. It can be observed that LRS or *R*_c_ is not so stable at 5 μA as compared to 50 and 500 μA. This suggests that the filament size is smaller for lower current compliance as compared to higher current compliance. However, HRS maintains the same level at different CCs for more than 10^4^ s. Kund et al. [9] have studied the variation of LRS with varying CC ranging from 100 nA to 100 μA, leading to the fact that a large number of levels can be achieved. Figure 10a presents a resistive switching memory device using Ge_0.2_Se_0.8_ solid electrolytes in an Al/Cu/Ge_0.2_Se_0.8_/W structure which can be operated at a very low CC of 1 nA. The sweeping voltage direction is shown by arrows 1 to 2. Figure 10b shows the behavior of resistance states with CCs ranging from 1 nA to 1 mA for Ge_0.2_Se_0.8_ and Ge_0.5_Se_0.5_ devices. For both devices, LRS decreases with increasing CCs from 1 nA to 1 mA owing to filament diameter increase. At a CC of <10 μA, the LRS of the Ge_0.2_Se_0.8_ device is lower than that of the Ge_0.5_Se_0.5_ device. This suggests that Cu ions can be easily migrated through a higher Se content, which results in a large variation of HRS. However, the average HRS of Ge_0.2_Se_0.8_ devices decreases faster than that of Ge_0.5_Se_0.5_ devices. This indicates that the filament dissolution length of Ge_0.5_Se_0.5_ devices is larger than that of Ge_0.2_Se_0.8_ devices because of the thinner filament diameter or less overshoot effect. So resistive switching properties strongly depend on the compositional variations of Ge_x_Se_1 − *x*_ solid electrolytes. However, the SET voltage, endurance, data retention, and so on may be controlled using different compositions of GeSe_*x*_ solid electrolytes as per the requirement of the applications.

#### Oxide-based materials

**Figure 11:**
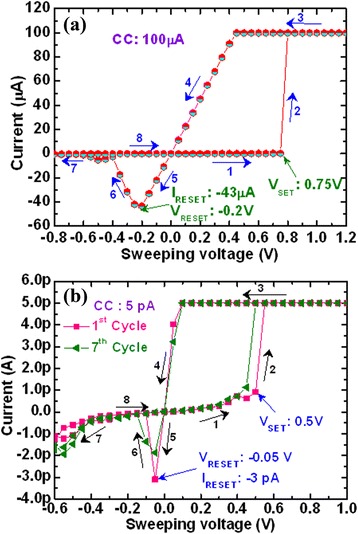
*I*-*V* hysteresis characteristics of a Cu/Ta_2_O_5_/W memory device. **(a)** Bipolar *I*-*V* hysteresis characteristics of a Cu/Ta_2_O_5_/W memory device. The memory device performs under a small operating voltage of ±1 V. Current compliance is 100 μA. **(b)** *I*-*V* hysteresis characteristics with a low current compliance of 5 pA are also obtained [17].

It is observed that the chalcogenide-based CBRAM device shows great potential for next-generation non-volatile memory, but one of the discrepancies is compatibility with the CMOS process. To mitigate this issue, binary metal oxides like Ta_2_O_5_ [16,17], SiO_2_ [18], ZrO_2_ [19], GeO_*x*_ [20,21], and so on have been reported, and their memory characteristics are discussed here. Sakamoto et al. [43] have reported bipolar resistive switching behaviors using a Cu/Ta_2_O_5_/Pt structure at a CC of 100 μA. However, a large *I*_RESET_ of >1 mA was reported. In our previous study [17], we have reported bipolar resistive switching phenomena using an Al/Cu/TaO_*x*_/W structure at a CC of 100 μA and a small operating voltage of ±1 V, as shown in Figure 11a. The voltage sweep is shown by arrows 1 to 8. The value of *I*_RESET_ is approximately 43 μA, and it is good for application. The values of *V*_SET_ and *V*_RESET_ are 0.75 and −0.2 V, respectively. This device is operated at a very low CC of 5 pA (Figure 11b). The *I*_RESET_ is approximately 3 pA. Although the device shows switching such a low current at 5 pA, it is difficult for real application because the operation speed will be also slower too. So far, it is operated at the lowest current in the literature. Schindler et al. [18,44] have investigated *I*-*V* characteristics using W/Cu:SiO_2_/Cu and Ir/Cu:SiO_2_/Cu cells and low operation currents of 1 nA and 5 μA, respectively. Tsunoda et al. [45] have reported CBRAM phenomena using a Ag/TiO_2_/Pt structure at a CC of 100 μA. Statistical distribution of the HRS and LRS of Al/Cu/TaO_*x*_/W memory devices shows also to be good in our previous study [17]. The memory device maintains an acceptable resistance ratio of cycle-to-cycle as well as device-to-device. Liu et al. [46] have reported the improvement of device-to-device uniformity introducing Cu nanocrystals (NCs) in Ag/ZrO_2_/Cu-NC/Pt structures. The NCs guide conductive filament (CF) growth and reduce the randomness of the CF formation and rupture processes, leading to improved stability and uniformity of the memory devices. Li et al. [19] have reported good cumulative probability (device-to-device) of SET/RESET voltage and HRS/LRS in Au/ZrO_2_/Ag devices. Even though *I*-*V* switching has shown to be good with low operation current, P/E endurance and data retention are also important, which are discussed below.

**Figure 12:**
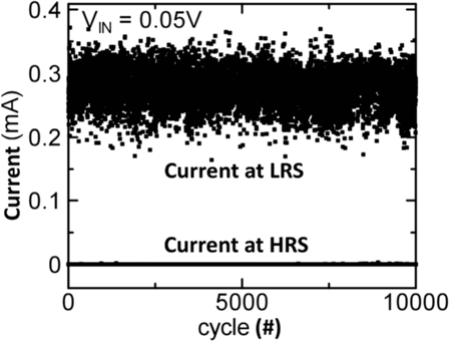
Program/erase endurance of >10^4^ cycles of a Ta_2_O_5_-based CBRAM cell. This shows good endurance; however, the operation current is also high [47].

**Figure 13:**
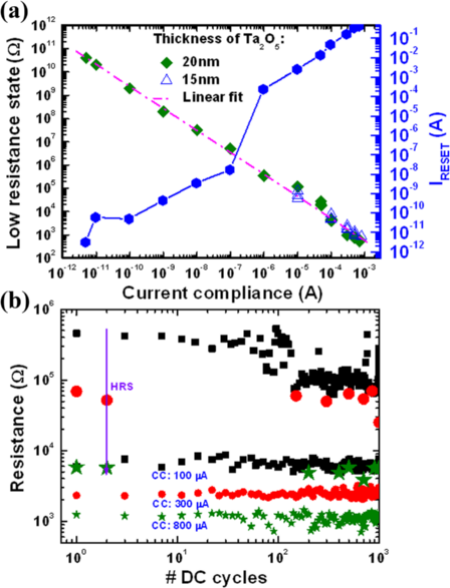
RESET current and DC switching cycles with current compliances. **(a)** RESET current (*I*_RESET_) with current compliances. The LRS decreases with increasing CCs. **(b)** DC switching cycles (>10^3^) with different current compliances. The thickness of the switching material is about 15 nm. A 100-Ω series resistance was used during endurance measurement. The device size is 1 × 1 μm^2^ [17].

Banno et al. [16] and Sakamoto et al. [47] have reported stable 10^4^ P/E cycles with 100-μs pulse width, as shown in Figure 12. Balakrishnan et al. [48] have reported >10^7^ P/E cycles in a Cu/SiO_2_/W CBRAM cell. To analyze the data retention of an oxide-based CBRAM device, Schindler et al. [18] have reported >10^5^ s data retention at a CC of 1 μA using a Cu/Cu:SiO_2_/W structure. Both HRS and LRS exhibit stable data retention with a large resistance ratio of >10^4^. Tsunoda et al. [45] have shown >10^4^ s data retention at a CC of 100 μA in a Ag/TiO_2_/Pt cell. We have reported good data retention at a CC of 50 μA of more than 200 h at 85°C with a high resistance ratio of >10^8^ using a Cu/GeO_*x*_/W structure [20]. Figure 13a shows the LRS vs current compliances. The LRS decreases with increasing CC from 5 pA to 700 μA, owing to the stronger Cu nanofilament formation in TaO_*x*_ films [17]. The value of LRS is independent of the thickness of TaO_*x*_ films. This suggests that our resistive switching memory device can be applicable for MLC. The RESET current increases with increasing CC from 5 pA to 700 μA, which indicates that a metallic filament can be formed by applying larger current. Figure 13b shows DC switching cycles, measured up to 1,000 cycles, with different CCs varying from 100 to 800 μA. The LRS decreases with increasing CCs because of the larger diameter of the filaments. However, the HRS decreases with increasing CCs, which is due to the remaining filament. This suggests that a single oxide-based switching layer has a cycle-to-cycle variation. Therefore, bilayer materials may have benefited to improve the switching cycles and can also maintain a high resistance ratio with demanded reliability too.

#### Bilayer materials

**Figure 14:**
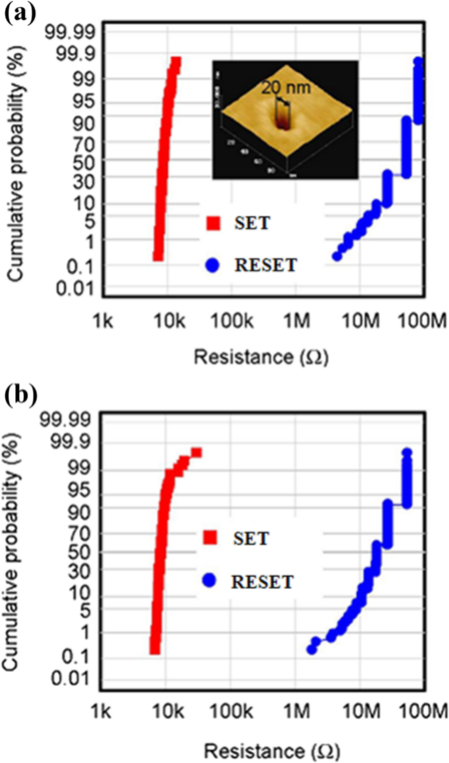
Statistical distribution of LRS under *V* > *V*_SET_ (SET condition) and HRS under *V* < *V*_RESET_ (RESET condition). **(a)** 20-nm (shown in the inset) and **(b)** 50-nm cell in Cu-Te/GdO_*x*_ CBRAM devices [24].

**Figure 15:**
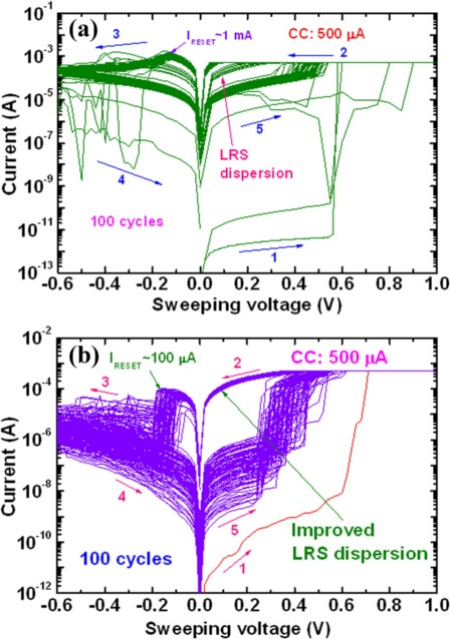
*I*-*V* characteristics of **(a)** Al/Cu/TaO_*x*_/W and **(b)** Al/Cu/TiO_2_/TaO_*x*_/W structures. The memory device shows excellent uniformity at LRS after introducing a Ti nanolayer in the Cu/TaO_*x*_ interface [29].

**Figure 16:**
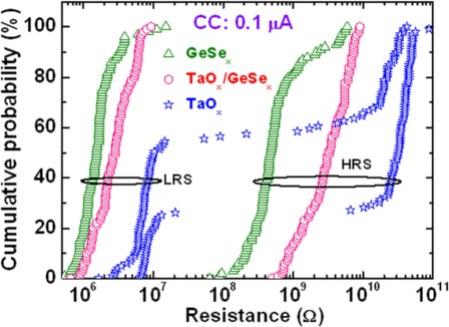
Cumulative probability of the LRS/HRS for the Al/Cu/GeSe_*x*_/W, Al/Cu/GeSe_*x*_/TaO_*x*_/W, and Al/Cu/TaO_*x*_/W CBRAM devices [28].

Several researchers have studied CBRAM phenomena using bilayer materials to control Cu or Ag diffusion and improve switching uniformity and other electrical performances as well. Different bilayers such as Cu-Te/GdO_*x*_ [24], MoO_*x*_/GdO_*x*_ [26], Ti/TaO_*x*_ [29], GeSe_*x*_/TaO_*x*_ [28], Cu-Te/Al_2_O_3_ [30], TiW/Al_2_O_3_ [31], and so on are discussed below. Figure 14 shows a device-to-device cumulative probability. Improved uniformity by using Cu-Te/GdO_*x*_ bilayer materials in 1T1R configuration for 20-nm (Figure 14a) and 50-nm (Figure 14b) cell sizes is reported [24]. A cell size of 20 nm in diameter is also observed by using an atomic force microscopy image, as shown in the inset of Figure 14a. No such inherent differences of LRS are found in between two different cell sizes, which represents that switching characteristics are insensitive to the device area and indicates the filamentary switching mechanism. By using MoO_*x*_/GdO_*x*_ bilayer materials, the *I*_RESET_ is reduced to 300 μA as compared to approximately 1 mA for a single MoO_*x*_ layer [26]. Improved switching characteristics by introducing a thin Ti nanolayer in Al/Cu/TiO_2_/TaO_*x*_/W structures have been observed by us [29]. Figure 15 shows the *I*-*V* switching characteristics of both resistive switching memory devices. One hundred consecutive switching cycles are indicated by arrows 1 → 5 under a CC of 500 μA. The memory device (Al/Cu/TaO_*x*_/W structure) shows a leakage current of approximately 1.5 pA at a *V*_read_ of +0.1 V for a pristine device (Figure 15a), lower than that of the current (23 pA) for the device in an Al/Cu/TiO_2_/TaO_*x*_/W structure (Figure 15b). Because of a higher charge-trapping density by introducing a Ti nanolayer, the average leakage current is higher than that of the device without a Ti nanolayer. The *I*_RESET_ of the Al/Cu/TiO_2_/TaO_*x*_/W devices is lower than that of the Al/Cu/TaO_*x*_/W devices (100 vs 1,000 μA). An improved LRS dispersion is also observed for Al/Cu/TiO_2_/TaO_*x*_/W devices owing to Cu migration controlled through a TiO_*x*_ nanolayer under external bias. A low operation current of 10 μA using a Cu/TiW/Al_2_O_3_/W structure is also reported by limiting the possible Cu diffusion and has shown resistive switching with limited variability [31]. The statistical distribution of HRS and LRS with different CCs of 25 and 10 μA is also reported. It can be observed that with reducing current compliance, the resistance ratio increases up to ten times, which may cause reduction of *I*_RESET_. This is attributed to the higher value of HRS and broadened memory window. An Al/GeSe_*x*_/TaO_*x*_/W device shows a low operation current of 100 nA [28]. Compared to those of pure GeSe_*x*_ in an Al/Cu/GeSe_*x*_/W structure and TaO_*x*_ in an Al/Cu/TaO_*x*_/W structure, improved uniformity of LRS/HRS with switching cycles is observed for GeSe_*x*_/TaO_*x*_ bilayer materials, as shown in Figure 16. Considering 100 switching cycles at a CC of 100 nA, average SET voltages are found to be +0.2, 0.28, and >0.3 V for GeSe_*x*_, TaO_*x*_/GeSe_*x*_, and TaO_*x*_ devices, respectively. A stable LRS/HRS of the GeSe_*x*_/TaO_*x*_ device is observed, as compared to that of pure GeSe_*x*_ and TaO_*x*_ devices. This is due to the Cu nanofilament formation in the TaO_*x*_ switching layer at the GeSe_*x*_/W interface. The LRS values are different because of defect control in switching materials. The GeSe_*x*_/TaO_*x*_ bilayer device can be operated with different CCs varying from 1 nA to 500 μA. The average (or standard deviation) values of the LRS for device-to-device are found to be 0.14 GΩ (4.7 × 10^7^), 5.2 kΩ (1.7 × 10^3^), and 601 Ω (117), and those of the HRS are 11.9 GΩ (9 × 10^9^), 0.34 GΩ (5 × 10^8^), and 9.6 MΩ (9.5 × 10^6^) at CCs of 1 nA, 50 μA, and 500 μA, respectively. The average resistance ratios are 85, 6.5 × 10^4^, and 1.6 × 10^4^ at CCs of 1 nA, 50 μA, and 500 μA, respectively. Due to such a high resistance ratio, this resistive switching memory device can be useful for MLC applications in the future with a wide range of CCs from 1 nA to 500 μA.

**Figure 17:**
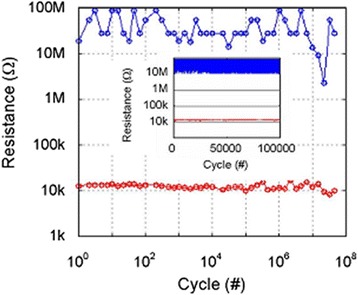
Pulse endurance of >10^7^ cycles of a Cu-Te/GdO_*x*_/W device. The pulse width was 10 ns. Every cycle up to 10^5^ is shown in the inset [24].

**Figure 18:**
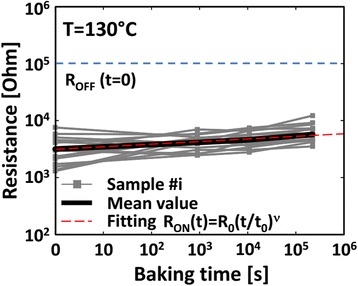
Good data retention characteristics of >10^5^ s at 130°C of a Cu:Ge-Te/Al_2_O_3_/TiN CBRAM device [49].

**Figure 19:**
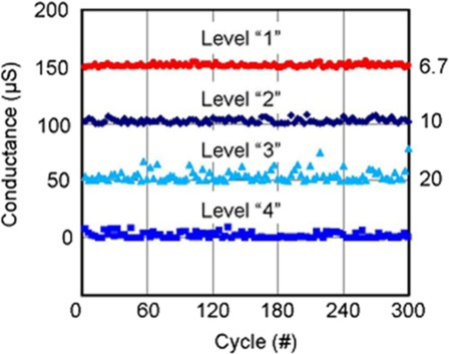
Four-level operations are shown by applying different operation currents. The first three levels are the SET with different programming currents, and the fourth level is the RESET condition. Pulse widths for SET/RESET are 10 ns/10 ns, respectively [24].

Introducing bilayer materials, long >10^7^ P/E cycles are reported, as shown in Figure 17 [24]. The programming current and pulse width are 100 μA and 5 ns, respectively. A large resistance ratio of approximately 100 is also shown in the inset. The programming current is also reduced to 25 μA [31]. A small pulse width of 10 ns is used. To review the retention behavior of a bilayer CBRAM cell, long data retention of >10^5^ s at 130°C in a Cu:Ge-Te/Al_2_O_3_/TiN CBRAM device with a small cell size of 20 nm is also reported [49]. Many devices are measured to observe the LRS values with elapsed time, as shown in Figure 18. A good resistance ratio of >10 is observed. Good data retention of >10^5^ s at 85°C under a small CC of 50 μA is reported [17]. Figure 19 represents the multi-level operation with variation of operation currents of Cu-Te/GdO_*x*_ bilayer materials [24]. Levels 1 to 4 are observed clearly. This depends on different programming currents, which can be used for MLC. In addition, the *V*_SET_ and *V*_RESET_ voltages are also important to control the switching characteristics as well as power. The device is operated with a high speed of 10 ns for the SET and RESET. Table 2 presents the *V*_SET_ and *V*_RESET_ of CBRAM devices with different switching materials. This indicates that bilayer materials show more reasonable *V*_SET_ and *V*_RESET_ voltages for application and the operation voltage of CBRAM devices is very good (±2 V).

**Table 2:** **SET and RESET voltages (** ***V***_**SET**_ **and** ***V***_**RESET**_ **) for various switching materials**

**Reference**	**+** ***V***_**SET**_ **(V)**	**−** ***V***_**RESET**_ **(V)**
[9]	0.25	0.08
[10]	0.2	0.1
[11]	0.2	0.05
[12]	0.75	0.03
[13]	0.3	0.3
[14]	0.3	0.2
[16]	0.15	0.1
[17]	0.75	0.2
[18]	0.9	0.15
[20]	0.6	0.2
[23]	0.5	0.25
[24]	1.0	0.75
[26]	1.1	0.7
[29]	0.6	0.15
[30]	0.5	0.3
[31]	1.5	1.0
[33]	0.56	0.16
[42]	0.4	0.2
[46]	0.5	0.2
[49]	0.7	0.5

### Switching mechanism

**Figure 20:**
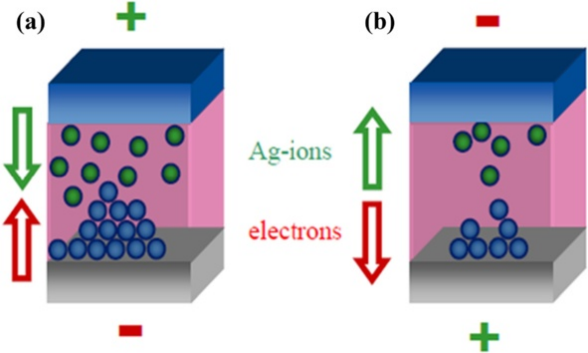
Simple schematic views of the devices at SET and RESET conditions. The device becomes **(a)** SET due to metallic path formation between the two electrodes, and **(b)** RESET is the result of the removal of the conductive path [50].

**Figure 21:**
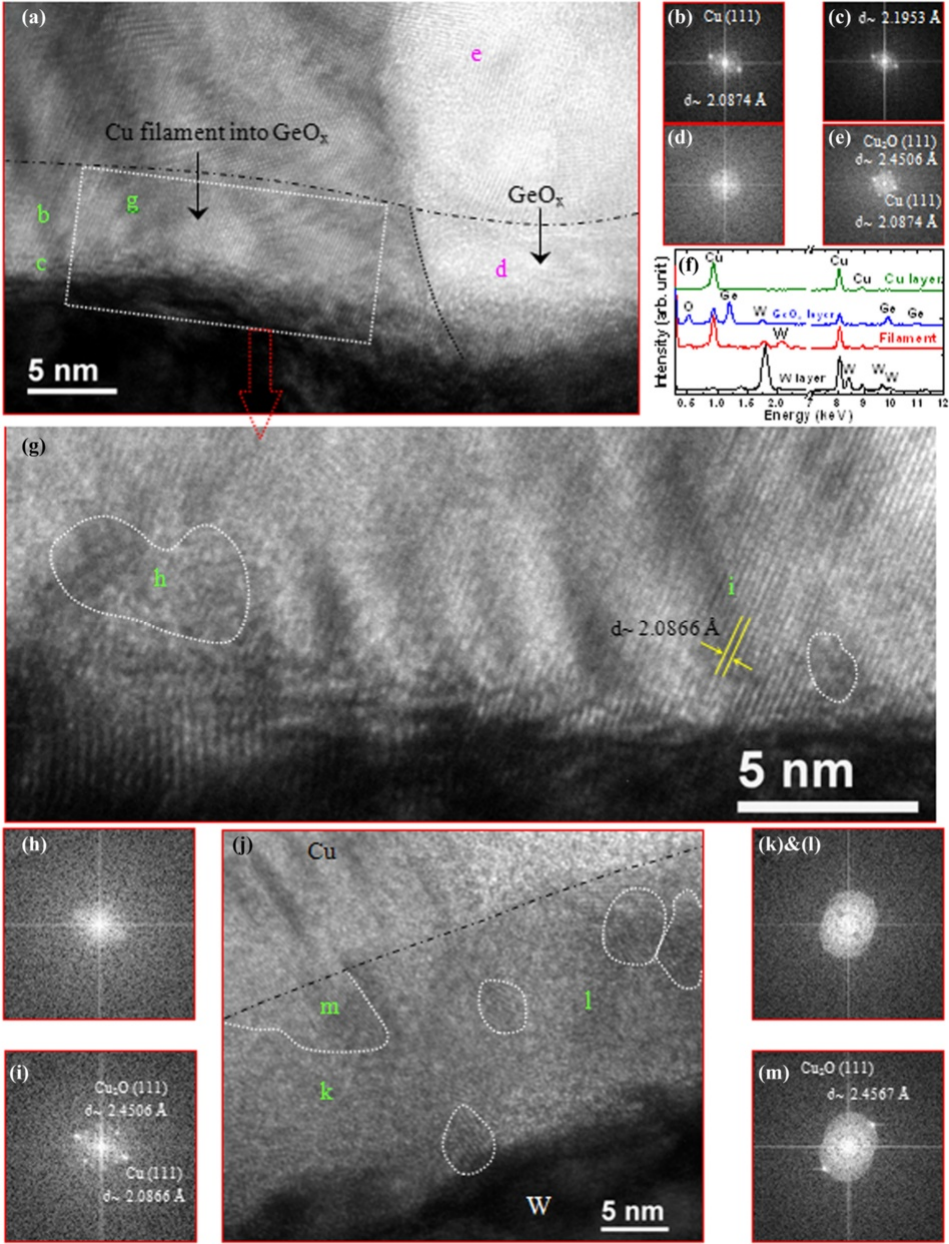
HRTEM images, FFT analysis, and EDX analysis of a Cu/GeO_*x*_/W memory structure. **(a)** After SET operation (CC of >100 μA), the HRTEM image of a Cu/GeO_*x*_/W memory structure. A crystalline Cu nanofilament is clearly observed in the GeO_*x*_ film. The FFTs confirm the **(b**,**g**,**i)** Cu nanofilaments, **(c)** Cu/W interface, **(d)** GeO_*x*_ layer without Cu nanofilament, and **(e)** Cu electrode. **(f)** All layers and filaments are also confirmed by EDX analysis. **(h)** Some amorphous regions are also observed. After the unipolar RESET operation, **(j)** an HRTEM image is shown. **(k**, **l)** An amorphous GeO_*x*_ layer with crystalline nanograins is observed because of the Ge-rich GeO_*x*_ film. **(m)** A small amount of Cu also remains on the Cu electrode because of higher Joule heating on the filament’s weak points [20].

Generally, it is known that the switching mechanism is based on the formation and removal of the metallic filament into the switching material, but there is one conflict about the growth kinetics of filaments. Therefore, filament formation/dissolution needs to be understood clearly for the mass production of CBRAM devices. When a positive bias is applied on the active electrode where the BE is grounded, then the Cu or Ag atoms are ionized and drift through the switching medium (solid electrolytes or oxide) under the electric field and finally reach the inert electrode. The filament formation/removal mechanism is reported first using a Ag_0.33_Ge_0.20_Se_0.47_ solid electrolyte material, as shown in Figure 20 [50]. Symanczyk et al. used Ag and Au as active and inert electrodes, respectively. Under positive bias on the Ag electrode (+*V* > *V*_SET_), Ag atoms become electrically oxidized and drift through the Ag_0.33_Ge_0.20_Se_0.47_ solid electrolyte and finally reduce at the Au BE due to incoming electrons from the BE. As a result, the metallic filament forms due to the gradual process and the memory device switches from HRS to LRS, which is known as the SET process (Figure 20a). Ionization equations for the redox (reduction and oxidation) reaction are as follows:

2 $$ \mathrm{Oxidation}:\mathrm{M}\to {\mathrm{M}}^{z+}+z{e}^{-},\ z=1,2 $$

3 $$ \mathrm{Reduction}:{\mathrm{M}}^{z+}+z{e}^{-}\to \mathrm{M} $$

where ‘M’ stands for Ag or Cu. When negative bias is applied on TE (−*V* < *V*_RESET_), Ag atoms in the filament become oxidized and migrate towards the TE, which results in the device to get back from LRS to HRS (Figure 20b). The resistive switching mechanism using a Ge_0.4_Se_0.6_ solid electrolyte in an Al/Cu/Ge_0.4_Se_0.6_/W structure was also investigated by us [12,33]. For HRS to LRS (+*V* > *V*_SET_), the Cu ions are generated at the Cu/Ge_0.4_Se_0.6_ interface from the Cu TE following the oxidation method as mentioned in Equation 2 and migrated towards the W BE due to the electric field, and then the Cu metal starts to grow from the W BE following the reduction method as mentioned in Equation 3 to form the Cu nanofilament in the Ge_*x*_Se_1 − *x*_ solid electrolyte through this redox reaction. It is reported that the base of the filament is at the cathode side and the thinner part of the filament at the anode side [33]. For LRS to HRS (−*V* < *V*_RESET_), the electric field will be higher at the Cu/filament interface and the Cu filament starts to dissolve at the Cu/Ge_*x*_Se_1 − *x*_ interface by Joule heating first and then the electrochemical oxidation at the top of the filament or the Ge_*x*_Se_1 − *x*_/filament interface. Basically, Joule heating at the weak part (i.e., the thinner part of a filament at the Cu/filament interface generally) of a metallic filament will play a role to dissolve the filament first. However, further study is necessary to understand the RESET mechanism. Then, the Cu ions are collected in the Cu electrode through the reduction method as mentioned in Equation 3. Therefore, the Cu filament length is gradually decreased (i.e., full removal of metals from the solid electrolyte), and the device is going back to pristine. It is interesting to note that the Cu ions can be migrated through porous regions in the solid electrolyte or defects. Therefore, defective solid electrolyte or oxide materials can be used to get such a resistive switching behavior. The Cu nanofilament is observed in Cu/Ge_0.4_Se_0.6_/W devices by TEM [33]. The device was prepared for TEM observation. Before going for TEM observation, the memory device was characterized with a program/erase of 4,000 cycles. Finally, the memory device was kept in a SET condition. The P/E current and pulse width applied are 500 μA and 500 μs, respectively. A huge Cu migration and filament formation are clearly observed. The Ge_0.4_Se_0.6_ film in the filament region shows to be crystalline owing to Cu ion migration and Cu filament formation after the SET condition. The diameter of the Cu nanofilament is approximately 11 nm. Some plausible explanations of Cu diffusion have also been discussed from other reported results in the literature. The diffusion occurs when copper or silver is in contact with chalcogenides, as reported by McHardy et al. [51]. The Cu or Ag reacts at room temperature with amorphous chalcogenides without any exposure to ultraviolet or electron radiation. After a few days, they have observed that copper diffuses throughout the GeSe_2_ film and polycrystalline materials such as Cu_2_Se (FCC) formed as small crystallites surrounding the amorphous film. In contrast, we observed that the Ge_0.4_Se_0.6_ film shows to be amorphous, which suggests that there is no diffusion of Cu initially unless external bias is applied. By applying external bias, structural changes (crystalline) occur in an amorphous Ge_0.4_Se_0.6_ solid electrolyte. A huge amount of Cu signal is obtained into the crystalline region, due to the formation of a Cu cluster or Cu nanofilament into the Ge_0.4_Se_0.6_ solid electrolyte. There are mainly two diffusion mechanisms involved: one is a fast interstitial mechanism and the other is a slow substitutional mechanism. However, Cu or Ag exhibits high ionic mobility in chalcogenides; therefore, Ag or Cu ions diffuse rapidly as interstitial species through these chalcogenides until a vacant site is encountered where substitution can occur [51,52]. Therefore, the defects play an important role for the formation/dissolution of a Cu filament through the solid electrolyte. Schindler et al. [53] have also reported the formation of Cu clusters on the surface of a Ge_0.3_Se_0.7_ film after deposition of a 160-nm Ge_0.3_Se_0.7_ film on a 50-nm-thick Cu layer. They also observed a Ag filament with a diameter of 20 nm in the Ge_0.3_Se_0.7_ solid electrolyte by conductive atomic force microscopy (CAFM) [53]. The Cu nanofilament was also observed by HRTEM after keeping the memory device at SET (Figure 21 [20]). A typical device size was approximately 150 × 150 nm^2^. The crystalline Cu nanofilament and GeO_*x*_ film (without Cu nanofilament) are shown. The clear crystallization in the GeO_*x*_ film shows a truncated conically shaped Cu nanofilament, which was also confirmed by EDX analysis (Figure 21f). The Cu signal calculated at the Cu electrode, GeO_*x*_ without filament, and Cu nanofilament in the GeO_*x*_ film are approximately 350, 200, and 400 at a typical energy of 0.92 keV, respectively. It is thus clear that the Cu signal is higher (400) in the Cu nanofilament than it would be without the Cu nanofilament in the GeO_*x*_ film. This nanofilament has Cu clusters (or nanocrystals) embedded in the GeO_*x*_ film or it is a Cu:GeO_*x*_ mixture. The Cu clusters are also confirmed by fast Fourier transform (FFT) analysis (Figure 21b,c,i), while the GeO_*x*_ film without Cu signals is shown in Figure 21d,h. Owing to the small crystals (nanograins) or amorphous GeO_*x*_ film, the FFT does not show a clear signal. Crystal *d*-spacing values of 2.0874 Å (Cu) and 2.4506 Å (Cu_2_O) are observed inside the nanofilament (Figure 21b,i), which are very close to the *d*-spacing value of pure Cu (111) of 2.0880 Å [28] and similar to that of the Cu electrode (Figure 21e). The *d*-spacing value of 2.1953 Å was observed at the Cu filament/W interface, which is in between the *d*-spacing value of Cu (111) and W (110) and was approximately 2.2378 Å (Figure 21c). A truncated conically shaped crystalline Cu nanofilament, the widths of the base and top being 85 and 70 nm, respectively, was observed. After unipolar RESET, a small crystalline GeO_*x*_ film is also observed (Figure 21j). Further, small crystals (3- to 5-nm grains), an amorphous GeO_*x*_ film (Figure 21k,l), and the remaining Cu on the Cu electrode (Figure 21m) are also found. This suggests that the Cu nanofilament has been dissolved almost completely after the RESET of the unipolar resistive switching mode, and thus, the device starts to behave as a pristine device. However, understanding of the growth/dissolution kinetics of the metallic filament under bias is also important.

**Figure 22:**
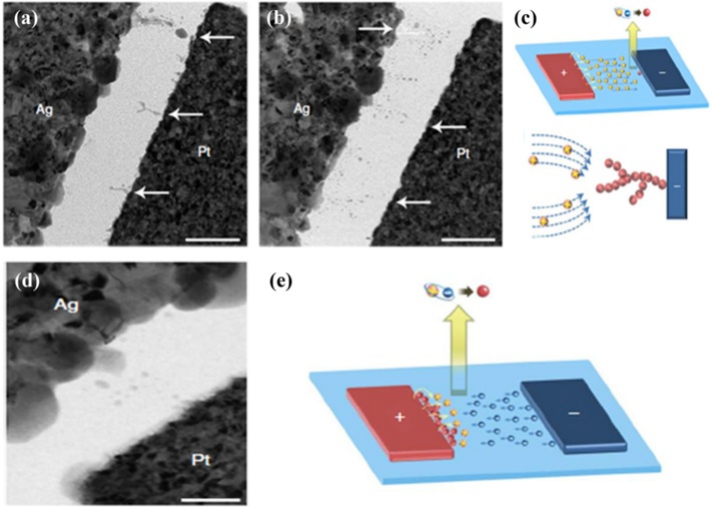
TEM images and schematic diagrams for the conducting filament kinetics. **(a)** TEM image of a SiO_2_-based material after the formation of the conducting nanofilament. The arrows indicate complete and incomplete nanofilaments. The scale bar is 200 nm. **(b)** TEM image after RESET operation. **(c)** Schematic illustration of the filament growth process. **(d)** TEM image of a conducting filament in a-Si-based CBRAM devices. The scale bar is 50 nm. **(e)** Schematic diagram of the nanofilament growth process in an a-Si CBRAM device [54].

Yang et al. [54] have reported the growth kinetics of nanofilament formation using a Ag/SiO_2_/Pt CBRAM device (Figure 22). When a positive voltage is applied to the Ag electrode, the Ag ion starts to inject into the SiO_2_ dielectric layer and forms a well-defined conducting filament with a typical conical shape highlighted by the top arrow in Figure 22a. It is interesting to note that the thinnest part of the filament is apparently near the inert Pt electrode interface, with a much wider base that formed near the active Ag electrode which is contrary to solid electrolyte-based cells. When negative bias is applied on the TE, it has been observed that the filament at the narrowest region near the filament/inert electrode interface starts to dissolve and the rest of the filament is no longer electrically connected to the positively biased inert electrode and device switched to HRS, as shown in Figure 22b. Since the cation mobility inside the solid electrolyte-based CBRAM devices is higher, a large number of cations become reduced and the filament starts to grow from the inert electrode. Hence, a dendrite-like filament with the base at the active electrode and the thinnest part at the dielectric/inert electrode interface is formed, as presented in Figure 22c. To further investigate the role of cation transport in dielectric films, they have chosen a-Si as the switching medium due to lower cation mobility compared with a SiO_2_-based device. When positive bias is applied on the TE, the filament starts to grow from the TE and a conducting path is formed in between two electrodes, as depicted in Figure 22d. This is due to very slow cation mobility through the a-Si by which before reaching to the inert electrode cations become reduced by the highly mobile incoming electrons from the inert electrode, as presented in Figure 22e. To unfold the switching mechanism of CBRAM devices, the exact filament dynamics is also very important, because the switching stability of the device depends on the filament geometry. So it is reported that the devices are initially at HRS or pristine, and after applying the external bias, the devices switch to LRS. This type of CBRAM device is gapless. On the other hand, there is another type of CBRAM devices where the devices are at LRS initially. This is known as gap-type atomic switch, whose switching mechanism is discussed below.

**Figure 23:**
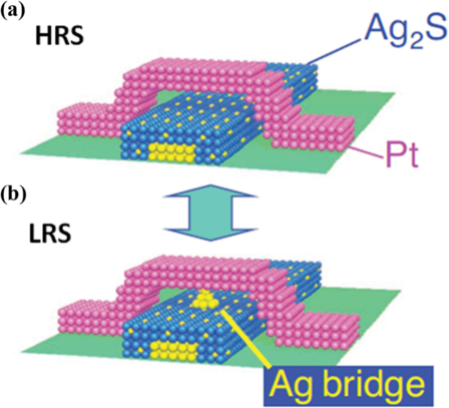
Schematic view of a crossbar structured for gap-type atomic switch. **(a)** A positive bias applied on the Pt nanowire makes the device in HRS by forming a 1-nm gap between the two electrodes. **(b)** A conducting Ag bridge is formed inside the gap by electrochemical reaction with the help of negative bias on the Pt nanowire [38].

**Figure 24:**
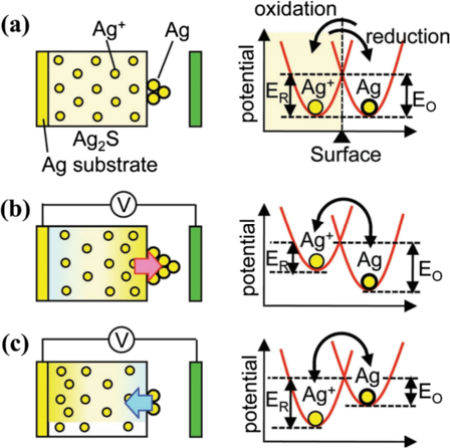
Simple schematic diagrams of the mechanism of Ag nanowire growth and dissolution process. **(a)** When no bias is applied, then the activation energies for reduction and oxidation are equal in the equilibrium condition. **(b)** Positive bias on the Ag_2_S electrode causes the diffusion of Ag^+^ cations towards the surface of the Ag_2_S electrode, making *E*_R_ smaller than *E*_O_. As a result, the reduction rate of Ag^+^ cations enhances. **(c)** Due to the negative bias on the Ag_2_S electrode, Ag^+^ cations diffuse towards the bottom of the Ag_2_S electrode, making *E*_O_ smaller than *E*_R_. As a result, Ag atoms oxidize easily and diffuse inside the Ag_2_S electrode [38].

**Figure 25:**
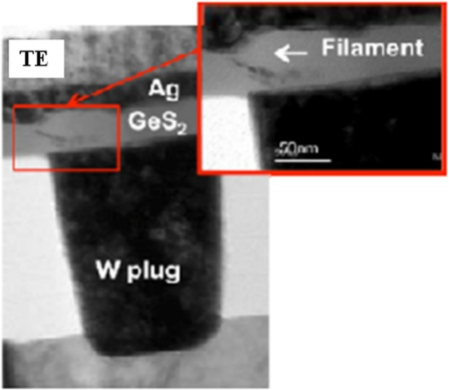
Cross-sectional TEM image of the memory device with a Ag/GeS_2_/W structure. The energy-filtered TEM image shows the existence of the Ag metallic path during the SET condition of the device [14].

Hasegawa et al. [38] have investigated an implicit mechanism of the gap-type switching phenomenon, as shown in Figure 23. Initially, the device is at LRS. Applying positive bias on a Pt nanowire, Ag atoms become Ag^+^ cations and diffuse through the Ag_2_S layer. As a result, forming a 1-nm gap, the device goes to HRS (Figure 23a). After reversing the polarity of the external bias, a nanobridge is formed inside the gap and the device switches to LRS (Figure 23b). These oxidation and reduction phenomena have been explained clearly by the schematic illustration shown in Figure 24 [38]. In the equilibrium condition when no bias is applied, the electrochemical potential of the Ag^+^ ions inside the Ag_2_S surface and that of the Ag atoms on the surface are equal. Hence, the activation energy of the oxidation (*E*_O_) and reduction (*E*_R_) remain the same, as shown in Figure 24a. When a positive bias is applied on the Ag substrate, Ag^+^ cations move towards the surface and hence the concentration of the Ag^+^ ions increases at the Ag_2_S surface. According to the formula of electrochemical potential, *μ* = *μ*^0^ + *Fφ* + *RT* ln*γC*, where *μ*^0^ is the potential for *γC* = 1, *F* is the Faraday constant, *φ* is the electrical potential, *R* is the molar gas constant, *T* is the absolute temperature, *γ* is the activity co-efficient, and *C* is the concentration of the metal cation, the potential of Ag^+^ ions increases due to the increase of the concentration resulting in *E*_R_ < *E*_O_, and the Ag nanowire starts to grow inside the gap (Figure 24b). Under negative bias, Ag^+^ ions migrate towards the bottom side of the Ag_2_S. As a result, the concentration of the Ag^+^ ion decreases at the sub-surface, resulting in *E*_o_ < *E*_R_, which enhances the oxidation rate of the Ag atoms and responsible for the HRS of the device (Figure 24c). Recently, Vianello et al. [14] have shown evidence of filamentary-based CBRAM operation using a Ag/Sb-doped GeS_2_/W structure (Figure 25), which can be used for proto-typical production. In the energy-filtered TEM image, it is clearly shown that a metallic filament is formed under SET condition, which makes a conducting path inside the solid electrolyte. Two parameters, temperature and voltage, play an important role for this process. The positive voltage on the Ag active electrode is responsible for the nucleation of the activated Ag ions which migrate across the solid electrolyte. As a result, a filamentary path is formed from the W inert BE electrode towards the active TE. On the other hand, a reverse bias causes the dissolution of the existing filament and results in the RESET of the device. Therefore, it is observed that the operation mechanism of CBRAM devices depends on not only the oxidation-reduction of the active electrode but also the several ways of filament formation dynamics which induce an important role on overall device performances. The switching mechanisms in different references of similar structures are always various, and they need to be unique from understanding point of view as well as application. Various mechanisms are observed which are owing to different switching materials, the anode/switching material interface, and different deposition methods. Therefore, selecting the switching material and deposition methods also has a major role. Further study is needed to explore the growth/dissolution kinetics of the metallic filaments in the future.

### Crossbar memory for 3D architecture

**Figure 26:**
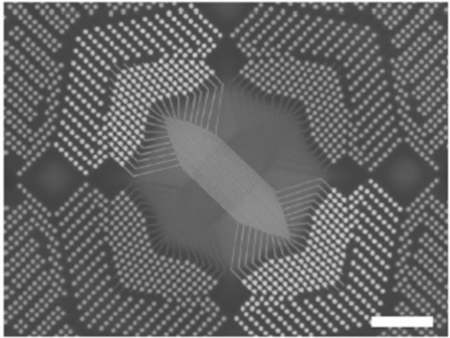
SEM image of a crossbar array fabricated on top of a CMOS chip [22]. This shows that high-density CBRAM devices could be produced.

**Figure 27:**
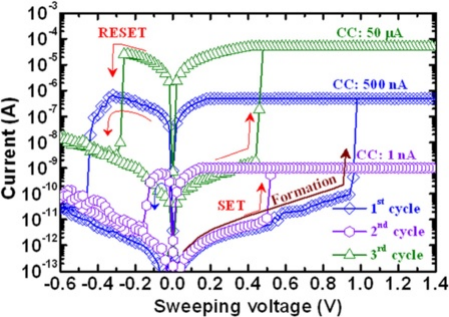
*I*-*V* switching characteristics of an Al/Cu/GeO_*x*_/W CBRAM device. The device can be operated at a low CC of 1 nA [21].

From the above discussions, it is concluded that CBRAM technology is the most efficient one for future nanoscale non-volatile memory application; however, one of the key issues is the necessity of high-density storage. Among the different types of device structures, the cross-point CBRAM device in 3D architecture is the most possible way to compete with multi-bit storage NAND FLASH. In addition, this crossbar memory can be used as a logic operation also [55]. Kim et al. [22] have demonstrated CBRAM switching using a Ag/a-Si/SiGe/W stack in 40 top nanowire electrodes crossed with 40 bottom nanowire electrodes in crossbar architecture, as shown in Figure 26. The operation of each cross-point of the integrated crossbar array is designed by a binary bitmap image with 1,600 pixels (40 × 40) representing data 0, i.e., the ‘HRS state’, and pixels representing data 1, i.e., the ‘LRS state’. For writing ‘1’ into a cell inside the array, a 3.5-V, 100-μs pulse is applied across the selected cell through the CMOS decoder circuit while the other unselected electrodes in the 40 × 40 arrays are connected to a protective voltage with amplitude equaling half of the programming voltage to minimize disturbance of the unselected cells. A similar approach is used for writing ‘0’ using a −1.75-V, 100-μs erase pulse. The programming/erasing is carried out based only on the input pattern and ignored the existing state of the memory cells, and a single programming/erase pulse is sufficient for each cell. Once all data are programmed in an array, the information in the array is then read out one cell at a time by applying a 1-V, 500-μs read pulse across the target cell, while grounding all unselected electrodes through the CMOS decoder. Stable switching with reasonable RESET current is needed to confirm the operations, which is not observed from their reports. Tada et al. [27] have also implemented a TiO_*x*_/TaSiO_*y*_ CBRAM stack in 4 × 4 crossbar architecture integrated with a CMOS circuit, and a higher operation current of >100 μA is also shown. According to our previous investigation [21], we have reported a cross-point CBRAM device using an Al/Cu/GeO_*x*_/W structure at a low current of 1 nA, as shown in Figure 27. Initially, all memory devices were in HRS. A formation voltage of >1 V is necessary to switch the memory device from HRS to LRS, which is shown in the first cycle and at a CC of 500 nA. After the formation process, the device shows normal bipolar resistive switching behavior. The memory device can be operated at a low CC of 1 nA, and a cylindrical-type filament can be expected because the HRS is the same after RESET operation. A change of HRS is observed at a CC of 50 μA. At a higher CC of 50 μA, the filament diameter is increased and the shape of the filament will be a conical type. After RESET operation, the Cu filament remains at the GeO_*x*_/W interface. On the other hand, a high formation voltage of approximately 6 V is needed for Al TE [21]. In this case, the memory device can be operated at a low CC of 1 nA, but a large RESET current of >1 mA is needed to rupture the oxygen vacancy conducting filaments. Further, Al TE will react with GeO_*x*_ and form an AlO_*x*_ layer at the Al TE/GeO_*x*_ interface. Using Al TE, a high *I*_RESET_ of >20 mA was also reported by Kato et al. [56]. Lin et al. [57] have also reported high *I*_RESET_ for Al_2_O_3_-based resistive memory using a Ti/Al_2_O_3_/Pt structure. According to several reported results, using an Al electrode is required for larger operation voltages as well as large RESET currents [56-58]. On the other hand, an excellent scaling of RESET current is observed for Al/Cu/GeO_*x*_/W cross-point memory devices with CCs from 1 nA to 50 μA. Furthermore, the RESET current is lower than the CC, which proves no current overshoot effect even 1R configuration or there is no parasitic effect. However, a GeO_*x*_ solid electrolyte-based cross-point memory in a Cu/GeO_*x*_/W structure shows a high resistance ratio of 10^8^ for future MLC applications. This suggests that the Cu nanofilament diameter can be controlled by CCs using Al/Cu/GeO_*x*_/W cross-point memory devices. Recently, a cross-point memory using an AlO_*x*_ switching layer also shows good characteristics for 3D architecture [59]. However, one of the key issues for 3D CBRAM storage is sneak path leakage. This can be solved by using a 1TnR or 1S1R (one selector-one resistor) structure. Jo et al. [60] have reported the integration of a 3D-stackable 1S1R passive crossbar memory. The selector shows a sharp switching slope of <5 mV/dec, selectivity of 10^10^, speed of <50 ns, and endurance of >10^8^ cycles. A 4-Mb 1S1R crossbar array shows that the sneak path leakage current is suppressed below 0.1 nA. It is expected that a high-density memory can be utilized with a low power for using CBRAM devices.

### CBRAM as a chip

**Figure 28:**
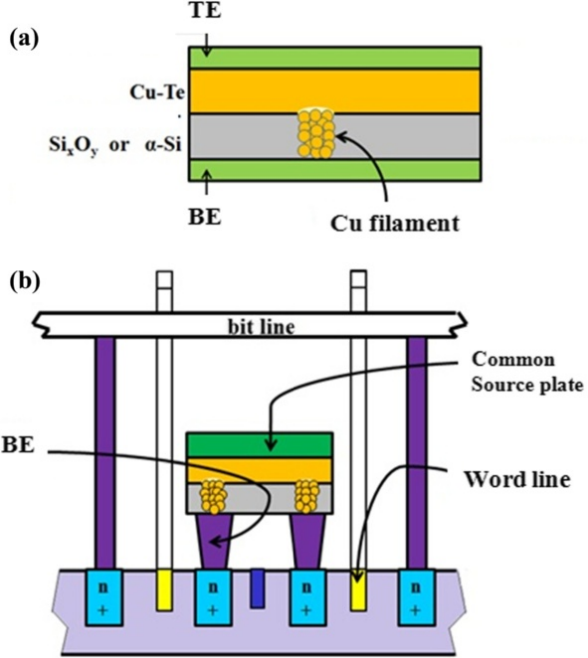
SET operation and dual cell of a CBRAM structure. **(a)** SET operation of a Si_*x*_O_*y*_-based CBRAM structure. **(b)** The dual cell of a CBRAM structure [64].

A CBRAM with a 32-kb chip has been reported by using I2C (Inter-Integrated Circuit) and SPI (Serial Peripheral Interface) bus architecture [61]. The operation voltage is approximately 2.5 to 3.6 V. Chevallier et al. [62] have reported a 64-Mb CBRAM storage device with a 130 nm technology node. Otsuka et al. [63] have reported a 4-Mb storage device in a 180 nm technology node with promising 2.3 GB/s read and 216 MB/s write throughput. Figure 28 shows a schematic diagram of dual-cell CBRAM devices using Cu-Te/SiO_*x*_ (or a-Si) layers [64]. The operating parameters of the 168-mm^2^ chips are the supply voltages of 1.2 and 5 V with an on-chip charge pump providing 6.6 V required for programming. It has two BEs which are driven by the two buried word line MOS transistors to write or erase the memory cells. The memory cell size is designed to be 6 F^2^. This storage device can be combined high performance and high bit density whereas its other counterparts are failing to achieve the same goal. Those CBRAM chips can be used as an economical alternative to the conventional Electrically Erasable Programmable Read-Only Memory (EEPROM) technology and may be applicable to electronic toys, games, consumer electronics, wireless LAN, data storage, etc. The concept of this dual cell is similar to that of complementary resistive switching [65], where two cells are in a series. It is expected that the CBRAM device can be mass productive in the near future, if there is a solution of reliability under few microampere operation and a clear understanding of the switching mechanism.

## Conclusions

In this article, we have reviewed CBRAM switching characteristics and switching mechanism, introducing chalcogenide, oxide, and bilayer switching layers. In the first section, we review the switching performance of chalcogenide-based CBRAM. It is observed that a chalcogenide-based memory device shows good bipolar resistive switching, long P/E endurance of >10^5^ cycles, and a good data retention of >10^5^ s at >85°C. The memory device also shows multi-level operation with varying current compliance from CCs of 5 to 500 μA. It is observed that the oxide-based memory device performs at a low CC of 5 pA, good device-to-device uniformity, good data retention at low current of 1 μA, and good P/E cycles as well. It is found that bilayer switching materials show excellent device-to-device uniformity, robust data retention, and better P/E cycles of >10^7^ with a high speed of few nanoseconds. The performances of CBRAM devices using different switching materials and structures are listed in Table 3. The switching mechanism is based on the formation and dissolution of the metallic filament depending upon electrical bias. The growth kinetics of the metallic path in different chalcogenide- and oxide-based switching materials are reported. However, it is still debated how to start growing/dissolving the metallic filament and which interface is responsible for different switching materials and structures also. It is suggested that further study is needed for the understanding of the switching mechanism. The cross-point memory in 3D architecture can have a promising solution for future high-density non-volatile memory with low power. CBRAM as a chip is showing to realize mass production in the future. However, the reliability issues in terms of stability of HRS, LRS, endurance, and data retention need to be solved and more studies are needed. It is expected that CBRAM devices have very good opportunity for scaled (<11 nm technology node) non-volatile memory devices or logic gate operations.

**Table 3:** **Performances of CBRAM devices**

**Device structure (TE/switching material)/BE**	**Operation current (μA)**	**Retention time (s)**	**P/E endurance (cycles)**
Ag/10% Sb-GeS_2_/W [14]	100	10^5^ at 150°C	>10^5^
Cu/Cu-doped SiO_2_/W [18]	5	10^5^ at 1 μA	10^7^
Cu/GeO_*x*_/W [20]	50	>10^6^ at 85°C	>10^3^
Cu/Cu-Te/GdO_*x*_/W [24]	110	3.6 × 10^5^ at 130°C	~10^7^
Al/Cu/Ti/TaO_*x*_/W [29]	0.1 to 300	10^4^	10^4^
Al/TiN/Cu/TiW/Al_2_O_3_/W [31]	25	6 × 10^2^ at 125°C	10^6^
